# Canine Leishmaniasis Progression is Associated with Vitamin D Deficiency

**DOI:** 10.1038/s41598-017-03662-4

**Published:** 2017-06-13

**Authors:** A. Rodriguez-Cortes, C. Martori, A. Martinez-Florez, A. Clop, M. Amills, J. Kubejko, J. Llull, J. M. Nadal, J. Alberola

**Affiliations:** 1 Departament de Farmacologia, Terapèutica i, Toxicologia, Facultat de Veterinaria, Universitat Autònoma de Barcelona, Bellaterra, 08193 Spain; 2 Center for Research in Agricultural Genomics (CSIC-IRTA-UAB-UB), Universitat Autònoma de Barcelona, Bellaterra, 08193 Spain; 3 Mon Veterinari, Passeig Ferrocarril 100, Manacor, 07500 Spain

## Abstract

The relationship between vitamin D deficiency and the risk of suffering from a plethora of health disorders, ranging from autoimmune processes to infectious diseases has been widely described. Nonetheless, the potential role of vitamin D in visceral leishmaniasis remains uncharacterized. In the Mediterranean basin, where the dog is leishmania’s main peri-domestic reservoir, control measures against the canine disease have shown beneficial effects on the incidence of human leishmaniasis. In this study, we measured the vitamin D levels in serum samples from a cohort of 68 healthy and disease dogs from a highly endemic area and we have also studied the relationship of these levels with parasitological and immunological parameters. The sick dogs presented significantly lower (*P* < 0.001) vitamin D levels (19.6 ng/mL) than their non-infected (31.8 ng/mL) and the asymptomatic counterparts (29.6 ng/mL). In addition, vitamin D deficiency correlated with several parameters linked to leishmaniasis progression. However, there was no correlation between vitamin D levels and the *Leishmania*-specific cellular immune response. Moreover, both the leishmanin skin test and the IFN-γ levels displayed negative correlations with serological, parasitological and clinical signs. Further studies to determine the functional role of vitamin D on the progression and control of canine leishmaniasis are needed.

## Introduction

Visceral leishmaniasis (VL) is a neglected disease provoked by protozoan parasites of the *Leishmania* genus. Mainly found in subtropical areas of Asia and Africa, Latin America, and Mediterranean basin, VL can be fatal if left untreated. 20,000 human deaths are attributed to VL annually (http://www.who.int/leishmaniasis/en/). The dog is the main peri-domestic reservoir of the etiological agent, the protozoan *L. infantum* and shares many clinico-pathological features with the human disease^1^. Most *Leishmania*-infected humans and dogs show an asymptomatic infection, with only 5–20% of the cases developing the patent disease over a variable period of time^2, 3^. Although the mechanisms that regulate the final outcome of the infection remain unknown, the current knowledge suggests that the protective responses are associated with the activation of specific cell-mediated immunity and a Th1-proinflamatory immune response^4^. Recent studies show that Th17 cells act synergistically with the Th1 population to control *L. infantum* growth. The interleukin (IL)-17 A modulates some key regulatory cytokines including IL-10 and sustains IFN-γ production by Th1cells at sites of inflammation^5^. The active disease, however, has been linked to high antibody levels and a progressive Th2-deactivating immune response in the presence of a strong inflammatory reaction^6^. Increasing evidences suggest that the innate immune system could play a relevant role by promoting the appropriate inflammatory response against the parasitic invasion^7^.

Vitamin D is the precursor of the powerful steroid hormone calcitriol. Besides its well described effects on the calcium and phosphate homeostasis, several studies strongly suggest that vitamin D has a strong immuno-modulatory role triggered by its binding to the vitamin D receptor (VDR), which is expressed in several antigen-presenting cells such as monocytes, macrophages, and dendritic cells^8, 9^. The mechanisms by which vitamin D modulates the immune function have been studied within different context involving multiple pathogens. One of the most profoundly investigated mechanisms involves the axis of low sunlight exposure, vitamin D deficiency, and tuberculosis, which this has been a subject of study for over a century^10^. Moreover, it is now known that vitamin D strengthens the innate immune system by inducing the expression of anti-microbial peptides, such as cathelicidin and β-defensin, in *Mycobacterium tuberculosis-*infected human macrophages^11^. These peptides carry out several anti-microbial functions by directly acting on the bacterial wall^12^, increasing the formation of reactive oxygen species, modulating cytokine expression^13^, and inducing autophagy, which is another relevant anti-microbial effector mechanism^14^. Recent studies have shown a direct effect of vitamin D on T and B cells, where it reduces the production of pro-inflammatory Th1 and Th17 derived cytokines^15, 16^, B-cell differentiation, and IgG secretion^17^. Vitamin D has also been linked as a risk factor of a plethora of autoimmune disorders, diabetes, cancer, and several infectious conditions such as toxoplasmosis, influenza, malaria, and acquired immune deficiency syndrome^18–23^. Interestingly, a recent cross-sectional study conducted in Ethiopia showed that more than half of the paediatric VL patients had vitamin D deficiency^24^.

The goal of the current study was to investigate whether vitamin D levels are associated with the progression of canine leishmaniasis (CanL). To this end, we aimed at determining the potential correlation between the levels of the stable circulating metabolite of vitamin D, calcifediol (25-hydroxyvitamin D; 25(OH)D) and the *Leishmania*-specific immune response and the parasitological status of both clinically healthy dogs and those suffering from the disease. In addition, we investigated the genetic association of a single nucleotide polymorphism in the *VDR* gene to CanL susceptibility.

## Results

### Characterization of the canine population

Eighteen of the 68 dogs included in the studywere negative for *Leishmania* serology, *Leishmania* quantitative real-time PCR (qPCR) in blood, and also for the leishmanin skin test (LST), and were thus included in the group of “Non-Infected” animals^25, 26^. The remaining 50 dogs tested positive for, at least, one of the above tests, and they were considered as infected. Of these, 35 were clinically asymptomatic and 15 showed clinical signs of leishmaniasis.

The baseline characteristics of the three groups are shown in Table 1. The group of dogs suffering from clinical leishmaniasis presented a median [interquartile range] clinico-pathological score of 13.0 [10.50–18.50], a value that is significantly above the equivalent measurement in the non-infected animals (*P* < 0.001). Their main clinical signs were cutaneous lesions and lymphoadenopathy accompanied by higher levels of β- and γ-globulins (*P* = 0.016, *P* < 0.001, respectively) and lower red blood cell counts (*P* < 0.001). On the other hand, the group of asymptomatic dogs showed a median clinico-pathological score of 3.0 [1.00–4.00], a value that was non- statistically different when compared to the non-infected cohort (2 [1.00–2.75]) (*P* = 0.240).

**Table 1:** Baseline characteristics of the dog population used in the current study.

PARAMETERS	GROUPS OF ANIMALS						REFERENCE RANGE
	Non-infected (N = 18)		Asymptomatic (N = 35)		Symptomatic (N = 15)		
	Median [IQR]	Positive dogs^2^	Median [IQR]	Positive dogs^2^	Median [IQR]	Positive dogs^2^	
**ALT (U/L)** ^**1**^	48.5 [36.0–99.8]	5 (28%)	42.0 [35.2–51.7]	1 (3%)	38.0 [29.5–82.5]	2 (13%)	**21–102**
**ALP (U/L** ^**1**^	43.3 [29.9–53.4]	0	35.2 [24.7–46.7]	0	54.0 [34.0–85.0]	2 (13%)	**20–156**
**Creatinine (mg/dL)**	1.0 [0.9–1.2]	0	1.1 [0.9–1.2]	0	1.0 [0.7–1.2]	1 (7%)	**0.5–1.5**
**Urea (mg/dL)**	44.4 [33.4–48.0]	0	47.9 [35.9–56.3]	0	47.2 [31.9–2.7]	2 (13%)	**21.4–59.9**
**Albumin (g/dL)**	3.3 [3.0–3.8]	1 (6%)	3.1 [2.8–3.3]^a^	2 (6%)	2.3 [1.5–2.6]^**a**^	11 (73%)	**2.6–3.3**
**Beta-globulin (g/dL)**	1.4 [1. —1.7]	6 (33%)	1.7 [1.5–2.0]^a^	2 (6%)	1.8 [1.6–2.1]^**a**^	11 (73%)	**0.9–1.6**
**Gamma-globulin (g/dL)**	0.6 [0.4–0.7]	1 (6%)	0.7 [0.6–0.8]	3 (9%)	2.5 [1.2–4.1]^**a**^	13 (87%)	**0.3–0.8**
**RBC (10^6 cells/uL)**	7.1 [6.5–7.6]	1 (6%)	6.8 [6.3–7.3]	0	5.1 [4.6–6.2]^**a**^	10 (66%)	**5.5–8.5**
**Platelets (10^3 cells/uL)**	296.0 [227.0–353.0]	2 (11%)	286.5 [230.5–337.5]	2 (57%)	407.5 [202.5–581.5]	4 (27%)	**200–500**
**Neutrophiles (10^3 cells/uL)**	6.7 [5.19–9.9]	4 (22%)	6.7 [5.4–7.9]	0	6.3 [4.5–7.6]	3 (20%)	**3–11.5**
**Lymphocytes (10^3 cells/uL)**	2.6 [1.8–3.4]	1 (46%)	2.6 [1.8–3.3]	1 (3%)	1.8 [1.2–2.3]^**a**^	2 (13%)	**1–4.8**
**LAP** ^**1**^	—	2 (11%)	—	8 (23%)	—	14 (93%)	—
**Cutaneous**	—	0	—	5 (14%)	—	14 (93%)	—
**Ocular signs**	—	0	—	0	—	6 (40%)	—
**Musculuatrophy**	—	0	—	1 (3%)	—	3 (20%)	—
**Urinary signs**	—	0	—	0	—	0	—
**GI** ^**1**^	—	0	—	0	—	0	—
**Lameness**	—	0	—	0	—	0	—
**Epistaxis**	—	0	—	0	—	0	—
**Anti** ***-Leishmania*** **Antibodies (EU)**	7.0 [4,29–12,98]	0	15.9 [7.76–38.17]^**a**^	16 (46%)	300.0 [193.85–300.00]^**a,b**^	15 (100%)	**<0.22**
**Parasite Load in blood (parasites/mL blood)**	0.0 [0.00–0.00]	0	0.0 [0.00–4.32]^**a**^	12 (34%)	12.4 [5.95–24.69]^**a,b**^	11 (73%)	**0**
**Leishmanin skin test reaction (mm)**	0.0 [0.00–0.00]	0	12.0 [0.00–22.75]^**a**^	23 (66%)	0.0 [0.00–0.00]^**b**^	2 (13%)	**<5**

a = Statistically different from non-infected dogs (*P* < 0.05); b = Statistically different from asymptomatic dogs (*P* < 0.05).

^1^ALT: Alanin-amino tranferase; ALP: Alkaline phosphatase; LAP: Lymphadenopathy; GI: Gastrointestinal signs.

^2^Number of dogs from each group that showed a value out of the reference range for each one of the parameters under evaluation.

The three groups also showed statistically significant differences in the *Leishmania*-specific immunoglobulin concentration, the parasite burden in blood, and the intensity of LST reactions (Table 1). The symptomatic animals developed a higher level of *Leishmania*-specific antibodies than the non-infected dogs (*P* < 0.001). Moreover, 73% of the sick dogs were positive to qPCR (Ct threshold 39) in blood whilst only 13% showed a positive LST reaction. In clear contrast, the asymptomatic dogs displayed a significantly weaker specific humoral response when compared to the disease group (*P* < 0.001). Moreover, 66% of the asymptomatic animals were LST positive, but only 34% presented detectable parasites in blood. We also measured *Leishmania*-specific cytokine production by peripheral blood cells. These results showed that the IFN-γ levels produced by asymptomatic dogs (615.3 [130.80–1579.00] pg/mL) were higher than those produced by symptomatic (32.7 [1.73–125.42] pg/mL) or non-infected dogs (67.3 [0.00–208.59] pg/mL) (*P* = 0.001, *P* = 0.004, respectively). There were no differences between non-infected and sick animals in their IFN- γ levels. Furthermore, the comparison of IL-10 production of the three groups of dogs was similar.

### Vitamin D levels and canine leishmaniasis

**Figure 1:**
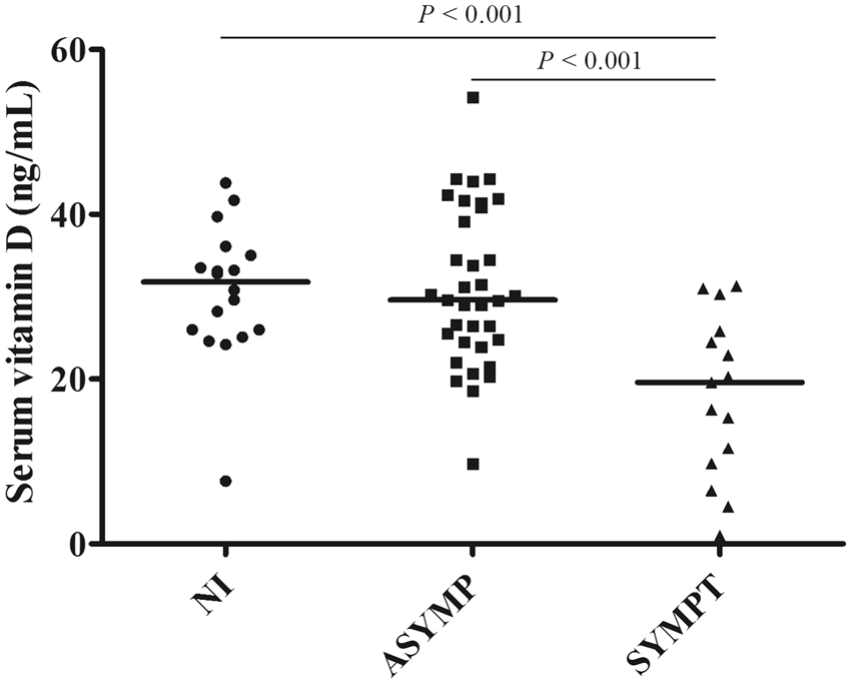
Serum vitamin D levels in dogs living in Mallorca and included in the study. Vitamin D status in dogs was assessed according to the serum levels of 25-hydroxyvitamin D (25(OH)D) estimated with an ELISA test. Non-infected dogs (NI), *Leishmania*-infected asymptomatic animals (ASYMP), and dogs clinically ill (SYMPT).

**Figure 2:**
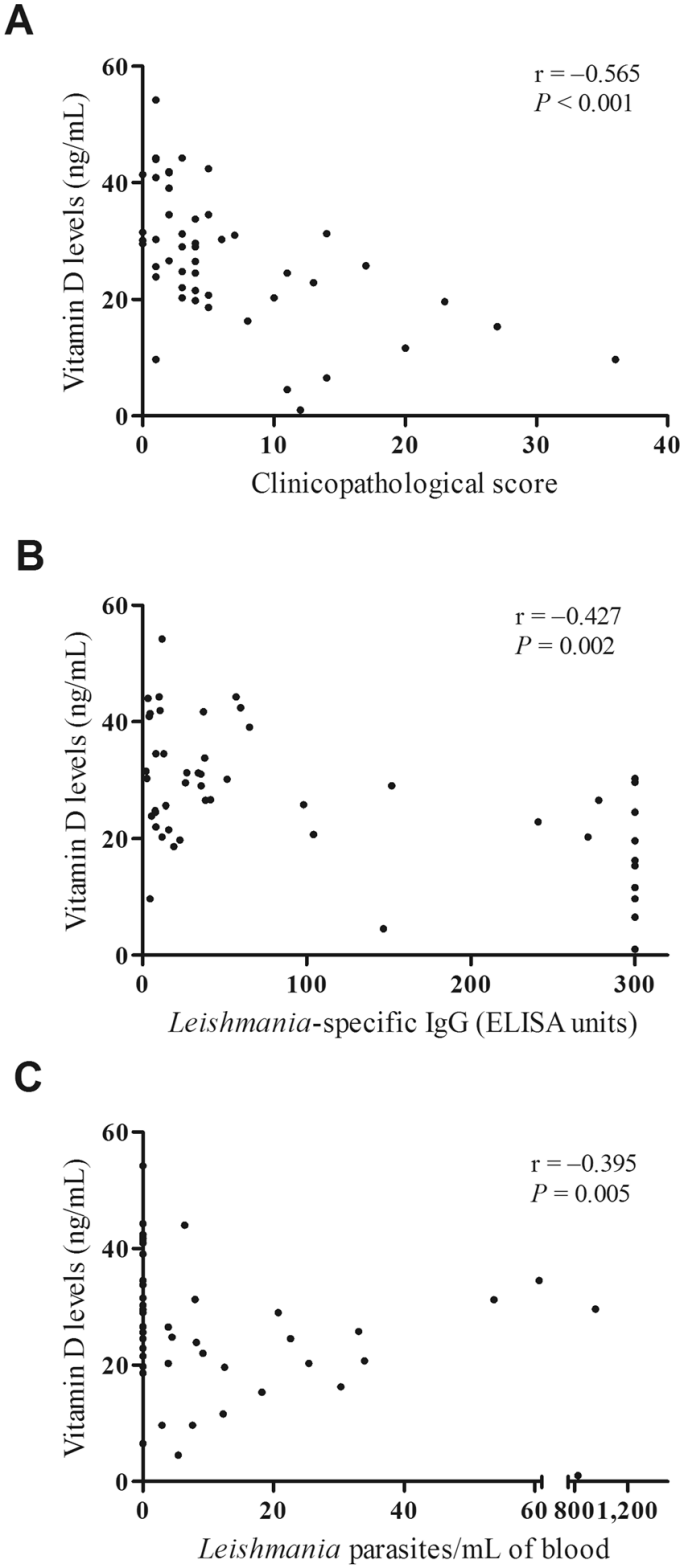
Correlations between vitamin D levels and clinicopathological score (**A**), *Leishmania*-specifc antibody levels (**B**) and parasite load in blood samples (**C**) from *Leishmania*-infected dogs. Spearman correlation indexes (r) and *P* < 0.05 are shown on the graphs.

As shown in Fig. 1, the dogs with patent disease presented significantly lower levels of 25(OH)D than the non-infected (*P* < 0.001) and the asymptomatic groups (*P* < 0.001). The median [interquartile range] levels of 25(OH)D in non-infected, asymptomatic, and symptomatic dogs were 31.8 [25.95–34.65], 29.6 [24.64–40.03], and 19.6 [10.62–25.14] ng/mL, respectively. In addition, vitamin D levels significantly correlated with the clinico-pathological score (*P* < 0.001), serology (*P* = 0.002), and parasite burden (*P* = 0.005) in blood samples (Fig. 2). We did not find a significant correlation between 25(OH)D levels and *Leishmania*-specific IFN-γ or IL-10 production. Whilst IL-10 was not correlated with any of the parameters under evaluation, the IFN-γ levels were positively correlated with LST reaction (ρ Spearman = 0.386, *P* = 0.008) and negatively correlated with the clinico-pathological score (ρ Spearman = −0.400, *P* = 0.006), serology (ρ Spearman = −0.469, *P* = 0.001) and parasite burden in blood (ρ Spearman = −0.369, *P* = 0.012).

Univariate logistic regression analysis showed that only vitamin D, IFN- γ, *Leishmania*-specific antibodies and LST reaction were significant explanatory variables. No association between canine leishmaniasis and age or sex was found. Table 2 shows the odds ratio (OR) calculated for every significant variable. Thus, for every unit (ng/mL) increase in plasma 25(OH)D, the risk of having leishmaniasis [(1-OR) * 100] declined by 14.09%. Likewise, for every one pg/mL increase in plasma IFNγ and every one mm increase in LST, the risk of having leishmaniasis declined by 0.24% and 14.11%, respectively. Finally, for every OD increase in ELISA CTLA, the risk of having leishmaniasis increased by 1.64%. In addition, according to the multivariate logistic regression model (Table 2), the increase of both 25(OH)D and LST was independently associated with a decreased risk of leishmaniasis by 21.45% and 28.73% per unit, respectively.

**Table 2:** Risk factors for canine leishmaniasis.

Univariate analysis	OR (CI 95%)
25(OH)D	0.8591 [0.7810–0.9451]
IFNγ	0.9976 [0.9954–0.9999]
ELISA CTLA	1.0164 [1.0086–1.0243]
LST	0.8589 [0.7687–0.9596]
**Multivariate analysis**	**OR (CI 95%)**
25(OH)D	0.7855 [0.6499–0.9490]
LST	0.7127 [0.5243–0.9689]

OR: Odds Ratio; CI: Confidence interval.

### Vitamin D receptor polymorphism

Vitamin D exerts its function by activating the VDR transcription factor. We therefore aimed to investigate whether genetic variation within the *VDR* gene locus is associated with the progression of CanL. We sequenced several genomic regions of the canine *VDR* gene (see Materials and Methods) and found four single nucleotide polymorphisms (SNPs), rs851938503 (A > G), rs852643282 (C > G), g.6894812 A > G and rs852900542 (T > C). Two of these variants were synonymous (rs851938503 and rs852643282) and mapped to exon 4 of the Ensembl’s (www.ensembl.org) annotated transcript ENSCAFT00000014506. The other two SNPs were located in introns 3 (g.6894812 A > G) and 7 (rs852900542). Genotyping of the 4 *VDR* SNPs in the cohort of 51 dogs showed intermediate allelic frequencies for SNPs g.6894812 A > G and rs851938503, and low minor allele frequencies (MAF) for SNPs rs852643282 and rs852900542 (Table 3 and Supplementary Table S2). Importantly, the allelic frequencies of the 4 SNPs were not statistically different between the three disease classes and between non-infected vs infected (symptomatic and asymptomatic) dogs, as determined with a χ^2^ test of independence (Table 4).

**Table 3:** Minor allele frequencies for each of the 4 vitamin D receptor SNPs genotyped in the study.

SNP name	MAF NI	MAF ASYMP	MAF SYMPT
rs851938503 (A > G)	0.40	0.33	0.32
rs852643282 (C > G)	0.03	0.08	0.05
g.6894812 A > G	0.40	0.35	0.36
rs852900542 (T > C)	0.10	0.10	0.05

MAF: Minor allele frequency; NI: non-infected dogs, ASYMP: the group of asymptomatic dogs, SYMPT: group of dogs suffering clinical leishmaniasis. In the SNP name column cells, the major and the minor alleles of the SNPs with an rs identifier are indicated between brackets.

**Table 4:** p-values of the χ^2^ test of independence comparing the allelic frequencies of the 4 SNPs among the phenotypic classes.

	g.6894812 A > G	rs851938503	rs852643282	rs852900542
Infected vs SYMPT	0.68	0.50	0.53	0.46
NI vs ASYMP	0.62	0.49	0.35	0.61
SYMPT vs ASYMP	0.77	0.69	0.45	0.42
NI vs Infected	0.76	0.48	0.45	0.72

NI: non-infected dogs, ASYMP: the group of asymptomatic dogs, SYMPT: group of dogs suffering clinical leishmaniasis.

## Discussion

In this study, we show the existence of a clear association between vitamin D levels and the outcome of VL. Indeed, 25(OH)D levels were significantly lower in dogs suffering from clinical leishmaniasis than in asymptomatic or non-infected animals living in the same area. In addition, vitamin D deficiency in *Leishmania*-infected dogs was strongly correlated with high parasite burden, and the low levels of this vitamin increased the odds of suffering from patent leishmaniasis. To the best of our knowledge, this is the first study that describes a statistically significant relationship between vitamin D deficiency and the progression of this parasitic disease. Low vitamin D levels in humans have been associated with a large number of diseases including multiple malignances, cardiovascular and metabolic disorders, autoimmunity and infectious diseases^18, 19, 27^. In veterinary medicine, low vitamin D levels in dogs have been recently linked with chronic enteropathy^28^, neoplasia^29^, and several infectious diseases^30, 31^. The establishment of associations between vitamin D levels and protozoan infections has yielded conflicting results in rodent models^20, 32–34^. However, recent studies have shed light on this issue by demonstrating a role for vitamin D in *Babesia* infections in dogs^30^, as well as in *Plasmodium falciparum* infection in children with vitamin D insufficiency associated with severe cerebral malaria in Uganda^21^. The oral administration of Vitamin D in rodents before or after *Plasmodium berguei* ANKA infection protected from cerebral malaria by dampening the systemic inflammatory response^35^. In addition, a recent study in Ethiopian children described vitamin D deficiency (<20 ng/mL) in more than half of the paediatric VL patients, although the robust of a relationship between vitamin D levels and clinical VL could not be established due to the lack of a healthy control group^24^. In contrast, patients with Post-Kala Azar dermal leishmaniasis— a clinical form characterized by high levels of anti-inflammatory IL-10 cytokine — presented a significant increase in vitamin D levels when compared to healthy controls^36^.

Due to the cross-sectional nature of this study, we cannot determine whether the low levels of vitamin D found in dogs with VL are the consequence or the cause of this parasitic disease. One hypothesis states that vitamin D deficiency in sick dogs could be due to an excess of vitamin consumption during the inflammatory process, following a similar pattern as the previously described for vitamin A during chickenpox infection in children^37^. Autier *et al*. (2014) performed a meta-analysis investigating the consequences of vitamin D supplementation in humans. They found that the raise of vitamin D levels did not alter the course of the group of health disorders that they studied and proposed that vitamin D deficiency is the result of inflammatory processes related to age, habits, and/or diseases^38^. One of the mechanisms that has been put forward to support this hypothesis is the rapid conversion of 25(OH)D to the bioactive 1,25(OH)_2_D by inflammatory cytokines that activate the enzyme catalyzing this reaction, CYP27B1^39^. Unfortunately, we could not analyze the 1,25(OH)_2_D levels in our canine population, as its half-life is very short and the test for its evaluation is only available at specialized laboratories.

An alternative hypothesis linking vitamin D and leishmaniasis is that dogs with vitamin D deficiency prior to infection might be at a higher risk of developing leishmaniasis. Although vitamin D levels have been determined in other canine infectious diseases^30, 31^, no longitudinal studies have been performed so far. The few longitudinal studies conducted in humans reported vitamin D deficiencies prior to clinical manifestations of multiple sclerosis^40^, pulmonary exacerbations in children with cystic fibrosis^41^, and more severe inflammatory bowel disease course^42^. Within the group of malignant neoplasias, colorectal cancer has been consistently associated with low pre-diagnostic vitamin D levels^43^. Both low UVB light exposure and low vitamin D-dietary intake have been suggested as the main causes of this prior vitamin D deficiency in humans. Nonetheless, dogs — unlike man — are unable to synthesize vitamin D from cholesterol precursors and UV skin irradiation^44^. In consequence, commercial pet food is supplemented, in a routine basis, with this ingredient to ensure that the vitamin D nutritional requirements are met. Two different scenarios could have caused the vitamin D deficiency that we observed in our dog population: either the diet of three groups was different or there were other factors involved in vitamin D storage/turnover affecting the serum 25(OH)D concentrations prior to disease. A longitudinal study would help resolving this relevant issue.

In any case, regardless of which scenario is more plausible, since vitamin D has a direct effect on the innate and adaptive immune response, a vitamin D deficiency, would with little doubt, have serious immunological consequences during the course of CanL. The activation of Toll-like receptors (TLR), a family of innate immune system receptors, triggers an antimicrobial response mediated by vitamin D. Indeed, the expression of *VDR* and *CYP27B1* are up-regulated in response to the activation of TLR-2 of human macrophages by *Mycobacterium tuberculosis* antigens^11^. Activation of TLR-2 in the presence of vitamin D increases the expression of target immunity genes, such as the anti-microbial peptide cathelicidin and β-defensin 4^45^. In the context of *Leishmania* infection, lipophosphoglycans and the cytosolic protein silent information regulator 2 from *L. infantum* activate TLR-2 in antigen presenting cells. In addition, the peptide BMAP-28 — a cathelicidin member family — has demonstrated anti-*Leishmania* effects and immuno-modulatory properties in *in vitro* cultures^46, 47^. These results would support the hypothesis of a beneficial role of vitamin D in innate immunity against *Leishmania* infection. In addition, vitamin D also exerts its immuno-modulatory role by shaping B cell and T cell responses. Exposing human B cells to 1,25(OH)_2_D inhibits their proliferation, IgG secretion andmemory B cell generation, and induces B cell apoptosis^17^. Although further studies to demonstrate the role of vitamin D on canine B cell immunity are needed, our results seem to tally with this finding, which may explain the strong inverse correlation between vitamin D and anti-*Leishmania* antibody levels detected in this work (Fig. 2B). On the other hand, there are several studies investigating the relationship between vitamin D and T cell immune response during the course of *Leishmania* infection. However, these studies focused on the cutaneous form in a rodent model and, in addition, they yielded discrepant results^32, 34, 48^. Our analysis did not show any association between *Leishmania*-specific IFN-γ or IL-10 production and vitamin D levels. As we have highlighted above, vitamin D function could related to the innate immune response rather than to adaptive immunity.

Remarkably, both the LST and IFN-γ levels displayed a negative correlation with the progression of leishmaniasis (clinical signs, serology and blood parasite burden). These results are in keeping with the importance that the Th1 immune response has in the control of this disease^4^. Moreover, our study finds a positive correlation between IFN-γ production and the LST reaction suggesting that a LST result could be a marker of IFN-γ status. There are few and poorly standardized assays that evaluate T-cell mediated immunity responses in dogs^49^. Whilst the techniques based on the detection of IFN-γ levels may represent an expensive and time-consuming technique, the LST test could be a field-adapted tool for the evaluation of *Leishmania*-specific cell-mediated immunity in epidemiological and vaccine-related studies.

Vitamin D activates the VDR transcription factor, which in turn, modulates the expression of several key genes involved in the immune response^50^. For this reason, genetic variation in the *VDR* gene changing its binding affinity, activity or protein levels may influence the immunological efficiency of vitamin D. In addition, *in vitro* and knock-out mice experiments have linked VDR to the resistance to *Leishmania major* infection^32^. Indeed, genetic variants in the *VDR* gene have also been associated with an increased risk of chronic chagasic cardiomyopathy^51^, malaria severity^52^, tuberculosis^53^, and some autoimmune disorders^54, 55^. Our results showed that the four *VDR* polymorphisms display similar frequencies across the non-infected, asymptomatic, and symptomatic dog groups (Table 3), and no genetic association between these SNPs and disease/infection status were observed (Table 4). The 4 SNPs that we investigated span a 10 kb genomic segment encompassing from intron 3 to intron 7 of the VDR transcript ENSCAFT00000014506. These SNPs are predicted to be either synonymous or intronic and should not alter protein function. They were screened as markers to identify any potential functional and causal polymorphism affecting VDR function. Moreover, two of our variants were present at low allelic frequencies in our population and this could have resulted in a low statistical power to detect a genetic association given the small sample size of our study. However, the canine *VDR* gene has 10 exons and spans a 58 kb long region, so we cannot rule out the hypothesis that other genetic variants elsewhere in the *VDR* gene affects the function or activity of VDR and, ultimately, impacts on the susceptibility to CanL infection. Moreover, a more comprehensive study involving additional genes from the vitamin D pathway such as *CYP2R1*, which converts vitamin D into 25(OH)D, would provide a deeper understanding of the relationship between genetic susceptibility to CanL and vitamin D metabolism. Larger studies involving more individuals and a higher density of genetic variants would increase the power to detect genetic associations in the vitamin D pathway to CanL susceptibility.

In summary, we report that progression of VL is strongly associated with vitamin D deficiency in dogs. Our findings suggest that vitamin D pathway may be involved in the immune response against *Leishmania* and that this may affect the susceptibility to suffer from the disease. A future goal would be to investigate if vitamin D supplementation may mitigate the symptoms and progression of this disease in infected dogs. Vitamin D supplementation could be a cost-effective and feasible strategy to be used both as an adjuvant therapy and to protect against the disease.

## Material and Methods

### Dogs and samples

All procedures were approved by the Universitat Autonoma de Barcelona’s Ethical Committee of Human and Animal Experimentation (Spain) in compliance with national (Royal Decree 1201/2005) and European Union regulations (European Directive 86/609/CE) for projects using animals for research purposes. Sixty-eight dogs attended at the Mon Veterinari Clinical Hospital of Manacor (Mallorca, Spain) were included in the study after their owners’ consent. The sample included 31 females and 38 males from different breeds, and ages ranged from 6 months to 15 years (4 ± 2.7 years). Prior to sampling, all dogs were examined for clinical signs compatible with CanL, such as weight loss, lymphadenopathy, cutaneous lesions, and ocular, gastrointestinal, and renal alterations. Blood samples were collected by jugular venipuncture. Serum was obtained after centrifugation at 3,000 rpm for 20 min, and stored at −20 °C until further use. Blood-EDTA samples used for *Leishmania* DNA detection were frozen at −20 °C before DNA extraction, and blood-heparin samples were analyzed before 24 h.

Clinical signs, biochemistry, and hematological values were scored as previously described with some modifications^56^. Briefly, clinical signs compatible with leishmaniasis such as cutaneous lesions, ocular signs, and epistaxis were scored 0–3 as indicated in Table S1. Both biochemistry and hematological results scored 1 point for each abnormal value (Table S1). These scores were summed up to obtain an overall clinico-pathological score for each dog. A score greater than 5 was considered as indicative of patent clinical leishmaniasis.

### Crude total *L. infantum* antigen (CTLA)-based ELISA

B cell function was analyzed by measuring anti-*Leishmania* antibody levels with an ELISA technique, as previously described^57^. Briefly, microtiter plates were coated with 2 µg of CTLA per well and sequentially incubated with sera and protein A conjugated to horseradish peroxidase (Pierce). Working dilutions were 1/400 and 1/15,000 for sera dilution and protein A-HRP dilutions, respectively. Absorbance values were read at 492 nm in an automatic microELISA reader (Anthos 2001, Anthos Labtec Instruments). Results were expressed in ELISA units (EU), referred to a known positive serum used as a calibrator, and arbitrarily set to 1 EU. Cut-off value (mean + 3 SD) for 76 dogs from a non-endemic area was set at 0.220 OD.

### Real-Time PCR amplification of *Leishmania* DNA in blood samples

Parasite load was determined by quantitative real-time PCR (qPCR) in blood samples. DNA was extracted using the High Pure PCR Template Kit (Roche). *Leishmania infantum* DNA was specifically detected and quantified with a TaqMan qPCR assay (Applied Biosystems). We employed a previously reported protocol^58^ with some modifications. The qPCR assay was designed to target a conserved DNA regions of the kinetoplast *L. infantum* genome. Primer sequences were LEISH-1 5′-AAC TTT TCT GGT CCT CCG GGT AG-3′, LEISH-2 5′-ACC CCC AGT TTC CCG CC-3′, and the TaqMan-MGB probe FAM-5′-AAA AAT GGG TGC AGA AAT-3′- MGB. The thermal cycling profile was 50 °C for 2 min, 95 °C for 10 min, 40 cycles at 95 °C for 15 seconds, and 60 °C for 1 min. Analyses were performed in a Step One Plus Real Time PCR System device (Applied Biosystems Laboratories). Each sample plus a negative control was analyzed in triplicate. The number of parasites per mL of blood was calculated using a standard curve generated with *L. infantum* DNA extracted from 1 × 10^7^ parasites by using serial dilutions from 10^3^ to 10^−3^ parasites. This technique was sensitive enough to detect 0.001 parasites per reaction with a dynamic range of 10^7^. The median slope of three different standard curves was −3.44, and the qPCR efficiency was 98%. Quantification was linear between 10^3^ and 10^−2^ parasites per reaction tube (correlation = 0.99).

### Leishmanin skin test (LST)

*In vivo* T cell-mediated immunity was determined in each dog by measuring delayed type hypersensitivity (DTH) response against the leishmanin reagent. The leishmanin reagent consisted of a suspension of 3 × 10^8^ inactivated *L. infantum* (MHOM/FR/78/LEM75) promastigotes per mL in a 0.4% phenol-saline solution. A volume of 0.1 mL of leishmanin solution was intradermally inoculated to dogs. The delayed-type hypersensitivity response was assessed by measuring the size of the indurated and erythematous area (mean of two perpendicular diameters) observed at 72 h post injection. A response against leishmanin reagent >5 mm was considered as an LST positive result^59^.

### Whole blood assay and cytokine detection

The *in vitro Leishmania*-specific immune response was evaluated by measuring pro-inflammatory (IFN-γ) and regulatory (IL-10) cytokine levels expressed by antigen stimulated lymphocytes in whole blood assays^60^. Blood collected in tubes containing heparin anticoagulant was diluted 1:10 in RPMI-1640 medium supplemented with 10% v/v heat-inactivated foetal calf serum, 10 mM Hepes buffer, 100 IU/mL penicillin, and 100 mg/mL streptomycin (Gibco, Paisley, UK). Cells were incubated in 96-well flat bottom plastic culture plates at 1.8 × 10^6^ cells per well with soluble *L. infantum* antigen (10 µg/mL), concanavalin A (2.5 µg/ mL), or media (unstimulated), for a period of 5 days at 37 °C in 5% CO_2_. Supernatants from each of the three replicate wells were pooled and stored at −80 °C.

Quantikine ELISA kits (R&D systems) were used to detect IFN-γ and IL-10 in supernatant cultures following manufacturer’s recommendations. Background levels in the non-stimulated control wells were extracted from the values on the antigen-stimulated wells to quantify the antigen-specific cytokine production.

### Determination of Vitamin D levels

Circulating levels of 25(OH)D are considered to be the most reliable estimate of overall vitamin D status because this is a stable circulating metabolite of vitamin D and its concentration is nearly 1,000-fold higher than that of 1,25(OH)_2_D_3_^27^. Thus, 25(OH)D levels were assessed in serum samples using a competitive direct enzyme-linked immuno-sorbent assay (IDS 25-Hydroxy Vitamin D Direct EIA kit, Immunodiagnostic Systems Ltd.) according to the manufacturer instructions and employing an automatic micro-ELISA reader (Anthos 2001, Anthos Labtec Instruments). The concentration of 25(OH)D in each sample was calculated using a four-parameter logistic curve fit (Graph Pad Prism v3.02), and results were expressed in ng/mL units. The threshold for 25(OH)D deficiency was set at <20 ng/mL (<50 nmol/L) as reported by Ross *et al*.^61^.

### Genetic variation at the Vitamin D receptor

With the aim to identify DNA variants in the *VDR* gene segregating in our dog population, primers targeting *VDR* exons 5, 8 and 9 and introns 4, 5, 7 and 8 were designed (Supplementary Figure S1). These regions were PCR amplified and sequenced in ten dogs. PCR reactions were prepared in a final volume of 15 µL containing 1.5 µL 10× PCR buffer, 0.25 mM of each dideoxynucleotide, 2.5 mM MgCl_2_, 0.3 µM of each primer, 0.75 U Amplitaq Gold DNA polymerase (ThermoFisher Scientific), and 6–22 ng of genomic DNA. Thermal cycling conditions included an initial step at 95 °C for 10 min, and 35 cycles of denaturation at 95 °C for 1 min, primer annealing at 62 °C (PCR amplicon 2) or 66 °C (PCR amplicon 1) for 1 min, and extension at 72 °C for 1 min. Subsequently, a final extension step at 72 °C for 7 min was carried out. Amplicons were purified with the ExoSAP-IT PCR cleanup kit (Affymetrix) and sequenced in both directions using the PCR primers and the Big Dye Terminator Cycle Sequencing Kit v1.1 (Applied Biosystems). Sequencing reactions were run in an ABI 3730 DNA Analyzer (Applied Biosystems) platform. Sequences were aligned with the SeqScape® v2.1.1..0 software (Applied Biosystems). Four polymorphic positions were identified and genotyped by direct sequencing of PCR products in a cohort of 51 samples including 15 non-infected, 25 asymptomatic, and 11 symptomatic dogs.

### Data analysis

In the unadjusted analysis, the comparisons between dog groups were performed using the Mann-Whitney *U* Test and the correlations between different parameters by Spearman’s rank correlation coefficient. We then adjusted for potential confounders between clinical leishmaniasis risk and vitamin D deficiency, considering clinical leishmaniasis as the dependent variable (outcome) and vitamin D levels, sex, age, serology, LST and cytokine levels as independent variables (predictors) using both univariate and multivariate regression analyses. To determine the genetic association of each polymorphism with the infection and disease status, the allelic frequencies of the three phenotypic groups were compared using the χ^2^ test for independence. All statistical tests were performed using SPSS version 15.0 (SPSS software, SPSS Inc). A *P*-value ≤ 0.05 was considered to be significant.

## Electronic supplementary material

Supplementary Figures and Tables
