# Supplementary material for: Canine Leishmaniasis Progression is Associated with Vitamin D Deficiency
Source: Sci Rep. 2017 Jun 13;7:3346. doi: 10.1038/s41598-017-03662-4 (PMC5469782; doi:10.1038/s41598-017-03662-4)
Supplement: Supplementary file 1 — Supplementary Figures and Tables [file 41598_2017_3662_MOESM1_ESM.pdf]

CANINE LEISHMANIASIS IS ASSOCIATED WITH VITAMIN D DEFICIENCY

Rodriguez-Cortes, A.[1*], Martori, C. [1], Martinez-Florez, A. [1], Clop, A. [2], Amills, M. [2], Kubejko, J. [2], Llull, J. [3], Nadal, JM. [3], Alberola, J[1*].

SUPPLEMENTARY FIGURES AND TABLES

FIGURE S1. Genomic location of the three amplicons used to identify and genotype genetic variation in the canine VDR gene.

a)

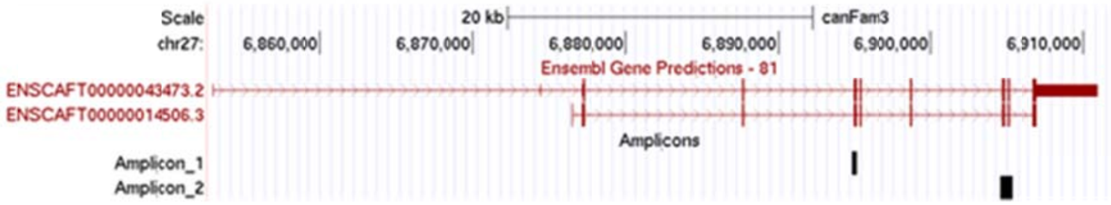

b)

amplicon	position	size (bp)	exons covered	introns covered
1	chr27:6,894,808-6,895,100	293	4	3,4
2	chr27:6,904,465-6,905,288	824	7,8	6,7,8

c)

amplicon	primer name	sequence
1	Cfa_VDR_amplicon_1_f	5´-ATCTGTTTGCCTTTAGCACCTG-3´
1	Cfa_VDR_amplicon_1_r	5'-CACCCCGCGGCTCATCGT-3´
2	Cfa_VDR_amplicon_2_f	5'-TGTCCAATCACAGCCTTGAG-3´
2	Cfa_VDR_amplicon_2_r	5'-GAGGGACCCCTCAACATACA-3'

The genomic coordinates are based on the dog genome CanFam3.1/canFam3 assembly.

a) Figure showing the location of the amplicons in relation to the canine VDR exons. The upper track displays the two known and annotated transcripts of this gene.

b) Table showing genomic location, amplicon size and the exons and introns (on the annotated transcript ENSCAFT00000014506) that are fully or partially covered by these amplicons.

c) Table showing the sequence of each primer from amplicon 1 and amplicon 2.

Table S1. Guide for scoring clinico-pathological signs compatible with canine leishmaniasis

Parameter	Alteration	Score
**Weight loss**	Normal (no weight loss or the animal grows normally)	0
	Weight loss <10%	1
	Weight loss between 10 and 20%	2
	Weigh loss >20%	3
**Cutaneous lesions (score for each cutaneous clinical sign: erythema, alopecia,...)**	Normal	0
	Slightly generalized (<10% of the body)	1
	Moderatly generalized (<25% of the body)	2
	Generalized (>25% of the body)	3
**Lymphoadenopathy**	Absence	0
	Localized	1
	Generalized	2
**Renal disorder**	Absence	0
	Poliuria	2
**Gastrointestinal disorder**	Absence	0
	Diarrhoea and/or emesis	2
**Splenomegaly**	Small increase on spleen size	3
	Large increase on spleen size	5
**Ocular**	Absence	0
	Slight and Unilateral	1
	Bilateral or severe unilateral	2
	Bilateral and severe	3
**Lameness**	Absence	0
	Localized	1
	Generalized	2
**Epistaxis**	Absence	0
	Presence	2
**RBC**	Normal	0
	Regenerative Anemia	1
	Non-regenerative anemia	2
**WBC**	Normal	0
	One hematological parameter is altered	1
	Two altered parameters	2
	3 altered parameters Consecutively	3
**Proteinogram**	Normal	0
	One parameter is altered	1
	Two parameters altered	2
	3 parameters altered Consecutively	3

Table S2. Genotype distribution for each SNP in the three phenotypic classes.

SNP	Genotype	Non-infected (n=15)	Symptomatic (n=11)	Asymptomatic (n=25)
g.6894812A>G	AA	5	4	11
g.6894812A>G	AG	8	1	10
g.6894812A>G	GG	2	2	4
rs851938503 (A>G)	AA	5	7	12
rs851938503 (A>G)	AG	8	1	9
rs851938503 (A>G)	GG	2	3	4
rs852643282 (C>G)	CC	14	10	21
rs852643282 (C>G)	CG	1	1	4
rs852643282 (C>G)	GG	0	0	0
rs852900542 (T>C)	TT	12	10	20
rs852900542 (T>C)	TC	3	1	5
rs852900542 (T>C)	CC	0	0	0
